# Effects of angular shift transformations between movements and their visual feedback on coordination in unimanual circling

**DOI:** 10.3389/fpsyg.2014.00693

**Published:** 2014-07-07

**Authors:** Martina Rieger, Sandra Dietrich, Wolfgang Prinz

**Affiliations:** ^1^Department of Psychology, Max Planck Institute for Human Cognitive and Brain SciencesLeipzig, Germany; ^2^Institute for Psychology, Department for Medical Sciences and Health Systems Management, University for Health Sciences, Medical Informatics and TechnologyHall in Tirol, Austria; ^3^Department of Education, Leipzig UniversityLeipzig, Germany

**Keywords:** unimanual coordination, visuo-motor transformation, angular shift, sensorimotor integration, tool transformation, circling, synchronization

## Abstract

Tool actions are characterized by a transformation between movements and their resulting consequences in the environment. This transformation has to be taken into account when tool actions are planned and executed. We investigated how angular shift transformations between circling movements and their visual feedback affect the coordination of this feedback with visual events in the environment. We used a task that required participants to coordinate the visual feedback of a circular hand movement (presented on the right side of a screen) with a circling stimulus (presented on the left side of a screen). Four stimulus-visual feedback relations were instructed: same or different rotations of stimulus and visual feedback, either in same or different y-directions. Visual speed was varied in three levels (0.8, 1, and 1.2 Hz). The movement-visual feedback relation was manipulated using eight angular shifts: (-180, -135, -90, -45, 0, 45, 90, and 135°). Participants were not able to perform the different rotation/different y-direction pattern, but instead fell into the different rotation/same y-direction pattern. The different rotation/same y-direction pattern and the same rotation/same y-direction pattern were performed equally well, performance was worse in the same rotation/different y-direction pattern. Best performance was observed with angular shifts 0 and -45° and performance declined with larger angular shifts. Further, performance was better with negative angular shifts than with positive angular shifts. Participants did not fully take the angular shift transformation into account: when the angular shifts were negative the visual feedback was more in advance, and when angular shifts were positive the visual feedback was less in advance of the stimulus than in 0° angular shift. In conclusion, the presence and the magnitude of angular shift transformations affect performance. Internal models do not fully take the shift transformation into account.

## INTRODUCTION

Tool actions are characterized by a transformation between movements and their resulting consequences in the environment. For instance, when pushing a lawn-mower movements result in consequences further ahead in the environment, or when pulling a sledge by a cord the consequences are behind the position of the actual movement. Transformations between movements and the consequences have to be taken into account when tool actions are planned and executed, and they are an important part of the cognitive representation of tool actions (Massen and Prinz, 2007). Some tool actions require the coordination of a tool’s consequences in the environment with other events in the environment. For instance, in baseball the tip of the baseball bat has to be coordinated with the position of the ball or in hockey the tip of the hockey stick needs to be coordinated with the position of the puck in order to achieve the intended goal. This is the topic of the present study: coordination performance when a transformation between a movement and its consequences exists.

Coordination principles have been studied using unimanual as well as bimanual tasks. In research on unimanual coordination (i.e., the coordination of one hand with an event or a stimulus) mostly tasks with discrete structuring events have been used. This is the case in tapping tasks (e.g., Aschersleben and Prinz, 1995), coincidence anticipation tasks (e.g., Fleury et al., 1992), and sometimes tracking tasks which include movement reversals (e.g., Alaerts et al., 2007). Research on bimanual coordination (i.e., the coordination of the two hands) has also included tasks without discrete structuring events, like circling (e.g., Swinnen et al., 1997; Mechsner et al., 2001; Tomatsu and Ohtsuki, 2005). Unimanual coordination of continuous movements in tasks without structuring events has rarely been investigated, and little is known how tool transformations affect coordination in such tasks (but see Dietrich et al., 2012; Rieger et al., 2014). Specifically, to the best of our knowledge it has not been investigated how the magnitude of an angular shift transformation between a movement and its visual feedback in the environment affects coordination performance in circling movements. Therefore, this was investigated in the present study.

Research on bimanual coordination has shown that coordination stability depends on (a) the relation between the hands in reference to movements toward and away from the body midline (we refer to this as the x-axis in the following) and (b) the relation between the hands in reference to movements toward or away from the body (we refer to this as the y-axis in the following). Coordination is more stable when the two hands move in opposite directions on the x-axis (one hand moves to the left and one hand moves to the right) than with any other type of movement pattern between the limbs (Swinnen et al., 1997; Swinnen and Wenderoth, 2004; Tomatsu and Ohtsuki, 2005). The second most stable mode is moving the two limbs into the same x-direction, (both hands move to the left and to the right at the same time, e.g., Swinnen et al., 1997). With reference to the y-axis, performance is most stable when the hands move in same y-directions (toward and away from the body at the same time), the second most stable mode is when the hands move in opposite y-directions (one hand is moving away and one hand is moving toward the body). However, with high frequencies movements in different y-directions often become instable, resulting in a transition to more stable same y-direction patterns (e.g., Swinnen and Wenderoth, 2004). All other coordination patterns are less stable (Haken et al., 1985; Tomatsu and Ohtsuki, 2005). Thus, the most stable coordination performance is obtained when movements of the hands have opposite x-directions, and same y-directions, i.e., mirror symmetric movements (Swinnen et al., 1997). These effects are ascribed to motor constraints (the way the central nervous system issues motor commands, Swinnen et al., 1997; Cardoso de Oliveira, 2002; Heuer et al., 2004; Salter et al., 2004), motor related feedback (kinesthesis and proprioception, Mechsner, 2004), visual feedback (Mechsner et al., 2001; Bogaerts et al., 2003; Mechsner, 2004; Tomatsu and Ohtsuki, 2005; Kovacs et al., 2010a,b), and cognitive constraints (Weigelt et al., 2007).

Coupling phenomena found in bimanual coordination are often similarly observed in unimanual coordination (e.g., Wimmers et al., 1992; Buekers et al., 2000). In unimanual coordination there is no second limb with which movements are coordinated, but rather a coordinative stimulus/event. As there can be no motor constraints related to the second hand moving, unimanual coordination depends on the perceptual characteristics of the movement feedback of the moving hand, which can be either visual and/or proprioceptive/kinesthetic. Studies indicate that coordination is predominantly governed by visual feedback in many situations (Buekers et al., 2000; Roerdink et al., 2005; Dietrich et al., 2012), even though proprioception/kinesthesis must also be taken into account (Wilson et al., 2005a,b; Dietrich et al., 2012). It also depends on the type of task whether visual or kinesthetic/proprioceptive information is more beneficial for unimanual coordination (Alaerts et al., 2007).

Transformed visual feedback has been experimentally deployed to study how motor related feedback (kinesthesis and proprioception) and visual feedback interact and contribute to coordination performance (e.g., Mechsner et al., 2001; Alaerts et al., 2007). In a task similar to the one we used in the present study participants were asked to coordinate the visual feedback of a circular hand movement with a circling stimulus in order to produce different visual patterns on the screen (Dietrich et al., 2012). Those visual patterns consisted of visual feedback and stimulus rotating in same or different directions and moving in same or different y-directions. To dissociate movements and the associated proprioceptive/kinesthetic feedback from visual feedback, participants performed the task under regular and transformed visual feedback (180° angular shift between movement and visual feedback on the screen). A 180° angular shift of the visual feedback implies that when stimulus and visual feedback have same y-directions, the y-direction of the hand movements is opposite to the y-directions of the stimulus and the visual feedback. However, when stimulus and visual feedback have different y- directions, the y-direction of the hand movement is opposite to the y-direction of the visual feedback, but corresponds to the y-direction of the stimulus. In this task, coordination occurred mainly in visual space, (similar data patterns with regular and transformed feedback, vision-to-stimulus coordination), but subtle effects of coordination in movement space were also observed (smaller differences between same and different y-directions in visual space with transformed feedback, movement-to-stimulus coordination). The presence of a transformation affected performance negatively.

In the present study we used a similar task. However, in contrast to Dietrich et al. (2012) we used a wider range of angular shift transformations between movements and the visual feedback on the screen, in order to disentangle effects of the presence of a transformation and the magnitude of a transformation on performance. Participants were asked to coordinate a visual feedback dot (produced by the participants’ movement and presented on the right side of a screen) with a continuously circling stimulus dot (presented on the left side of the screen). They were asked to produce four different patterns of the dots on the screen. Two aspects of the stimulus-visual feedback relation were varied. First, we varied the rotation direction which was either the same or different. The stimulus dot always moved clockwise. In one condition participants were asked to move counterclockwise (correspondingly the visual feedback dot also moved counterclockwise), therefore stimulus and visual feedback have different rotation directions (i.e., different directions on the x-axis). In another condition participants were asked to move clockwise, resulting in same rotations of stimulus and visual feedback (i.e., same directions on the x-axis). Second, the y-direction of the stimulus-visual feedback relation was varied. We asked participants to produce same y-directions and different y-directions of stimulus and visual feedback. Based on the study by Dietrich et al. (2012), in which a similar task was used, we expected that performance would be better when the coordinative pattern required same y-directions in visual space. We were further interested in whether we could replicate the previous finding that participants have difficulties performing the different rotation/different y-direction pattern.

The movement-visual feedback relation was transformed by using angular shift transformations. We used 0 and ±180° angular shifts as in the previous study, and in addition three positive angular shifts (45, 90, and 135°, visual feedback is ahead of the movement), and three negative angular shifts (-45, -90, and -135°, visual feedback lags behind the movement). This was done in order to investigate the influence of magnitude and direction (in advance or behind the hand movement) of the angular shift transformations on coordination performance. If only the pattern in visual space is important for unimanual coordination, the different angular shifts transforming the movement-visual feedback relation should have no effect on performance, i.e., the *accuracy* of performance should be equal for different angular shifts, and should depend only on instructed patterns in visual space. However, if it matters that a transformation modifies the movement-visual feedback relation, best performance should be observed with 0° angular shift and performance should be worse with all other angular shifts. The latter was expected based on previous results (Tomatsu and Ohtsuki, 2005; Wilson et al., 2005a; Dietrich et al., 2012; Rieger et al., 2014). Most importantly, we were interested in whether the magnitude of the transformation matters for performance. On the one hand, one could expect that all angular shifts which are not equal to 0° are performed equally well (or bad), because they all imply that movement and visual feedback do not match in angular position. On the other hand, this mismatch is more drastic in larger angular shifts than in smaller angular shifts. One may therefore expect that performance varies gradually, depending on the magnitude of the shift. The latter prediction would be in accordance with previous results on gain transformations (Rieger et al., 2014). However, even though the 180° angular shift is the most drastic one (visual feedback and movement are a maximal distance apart) it might be easier than smaller angular shifts. A similar effect is found in bimanual coordination, concerning the relation between hands. Opposite y-directions of the hands are (apart from same y-directions) more stable than other relations between the hands (Haken et al., 1985; Zanone and Kelso, 1992; Tomatsu and Ohtsuki, 2005). The difficulty of the movement-visual feedback relation (and/or movement stimulus relation) might follow similar principles as the difficulty of hand–hand relations. A particular benefit of the 180° angular shift condition might be observed in the different y-direction conditions: here stimulus and movement have the same y-direction, i.e., participants can rely on movement-to-stimulus coordination, which may benefit performance (see Dietrich et al., 2012).

In addition, we varied the speed of the stimulus dot in three levels, because previous studies have shown that coordination performance deteriorates with increasing speed (Kelso, 1984; Haken et al., 1985; Heuer, 1993; Byblow et al., 1995; Carson et al., 1997; Roerdink et al., 2005), especially under transformation conditions (e.g., Salter et al., 2004; Alaerts et al., 2007). Spontaneous switches from difficult to easy coordination patterns more likely occur with higher speed (e.g., Semjen et al., 1995). Therefore, we expected that performance would deteriorate with increasing speed.

In addition to accuracy of performance, we were interested in *how* participants perform the task. Specifically, we were interested in whether participants’ movement feedback is on the ideal position as instructed, or whether it systematically lags behind or is advance of (leads) that position. We assumed that the visual feedback dot would be in advance of the stimulus dot when no transformation is present. Such a lead was previously shown in a similar experimental setup with gain transformations (Rieger et al., 2014). This effect most likely occurred because movements were performed with the right (dominant) hand and the visual feedback was presented on the right side of the screen (as in the present task). In bimanual coordination the dominant hand usually shows a slight lead over the non-dominant hands (Treffner and Turvey, 1995), which seems to be due to attentional factors, because the lead of the dominant hand disappears when attention is directed to the non-dominant hand (Amazeen et al., 1997). However, this lead might be affected by the shift transformation, because the shift causes the visual feedback to lag behind or to be in advance of the movement, which needs to be compensated.

## MATERIALS AND METHODS

### PARTICIPANTS

Sixteen adults (nine female and seven male, aged 20–39 years, *M* = 25.6 years, SD = 3.6 years) took part in the experiment. All participants were right-handed according to the Edinburgh Handedness Inventory (Oldfield, 1971) and reported normal or corrected-to-normal vision. They were paid 7 euros/h to participate in a single session. Participants gave informed consent. The study was conducted in accordance with the Declaration of Helsinki and was approved by an ethics committee.

### APPARATUS AND STIMULI

The experiment was programmed using the C-language in a Microsoft DOS environment. Movements were recorded using a Wacom UD A3 writing pad (resolution: 500 pixels per cm, sampling rate 100 Hz), which was connected to the computer via a serial port and positioned on a desk in front of participants. Stimuli were presented on a 17 inch cathode ray tube monitor (screen refresh rate: 75 Hz, resolution: 800 × 600 pixels). The center of the screen was aligned with the midsagittal axis of the participant’s body and located 15 cm higher than and behind the writing pad. The background of the screen was black.

The stimulus was a white dot (diameter = 0.43 cm, stimulus dot), moving clockwise on a circular trajectory (radius = 4.32 cm). A second white dot (visual feedback dot, radius 0.43 cm) was controlled by a stylus for the writing pad, which participants held. The stylus was fixed inside a crank (radius 5 cm) and could only be moved in circles. The crank was fixed below a wooden board (15 cm above the writing pad), which also served to shield the hand from view. The center of the circular trajectory of the hand was positioned 10 cm to the right of the body midline. The distance between the centers of the stimulus trajectory and visual feedback trajectory on the screen was 17.27 cm. Participants sat on a height-adjustable chair; eye-screen distance was approximately 60 cm.

### PROCEDURE AND DESIGN

**FIGURE 1:**
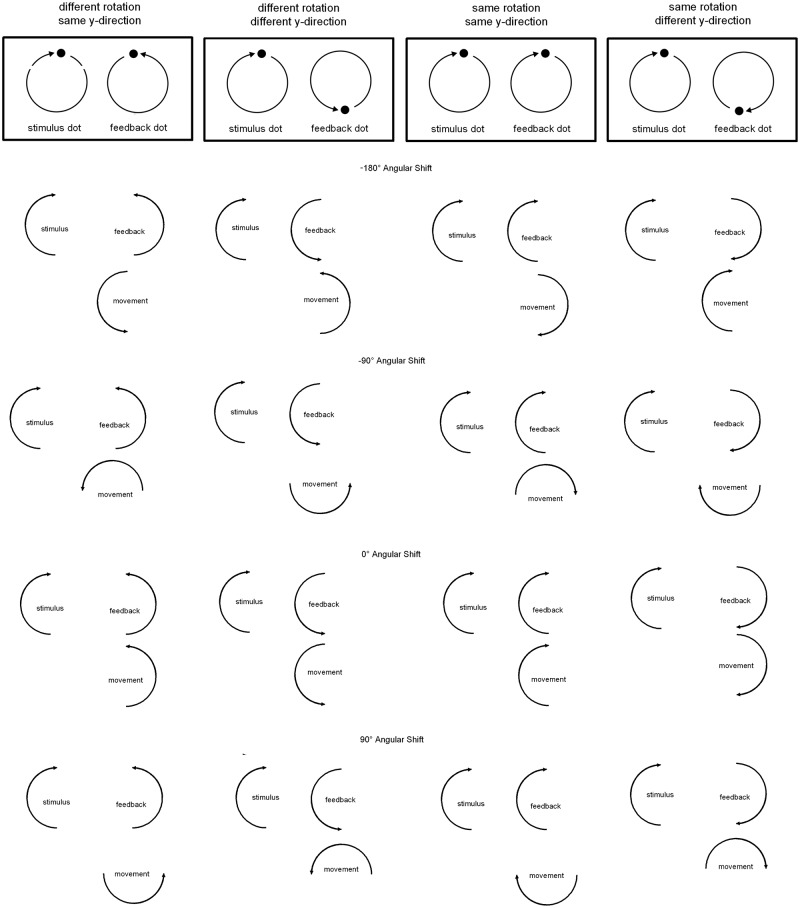
**Illustration of the four patterns participants were asked to produce (depending on rotation direction and y-direction) and examples of the angular shifts transforming the movement-visual feedback relation.** In the experiment eight different angular shifts were used: 0 and ±180° angular shifts, three positive angular shifts (45, 90, and 135°, visual feedback is ahead of the movement), and three negative angular shifts (-45, -90, and -135°, visual feedback lags behind the movement).

Participants were instructed on two characteristics of the visual patterns they were asked to produce. The first instruction concerned the rotation direction of the stimulus-visual feedback relation. Rotation direction could be the same, i.e., both dots moved clockwise, or different, i.e., the stimulus moved clockwise while the visual feedback dot moved counterclockwise. Second, participants were instructed on the y-directions of the stimulus-visual feedback relation. If y-direction was same, stimulus and visual feedback dots both moved upward and downward on the screen at the same time. If y-direction was different, the stimulus dot moved upward while the visual feedback dot moved downward and *vice versa*. An illustration of the patterns in visual space resulting from those instructions can be seen in **Figure 1**, upper part.

The movement-visual feedback relation was manipulated by introducing angular shift transformations. A certain angular value was added to (or deducted from) the hand position before being displayed on the screen. There were eight different angular shifts: 0 and ±180° angular shifts, three positive angular shifts (45, 90, and 135°, visual feedback is ahead of the movement), and three negative angular shifts (-45, -90, and -135°, visual feedback is behind the movement). For an illustration see **Figure 1**, lower part. Note that in the same rotation direction condition a positive angular shift meant that the visual feedback was shifted clockwise. In the different rotation direction condition a positive shift meant that visual feedback was shifted counter-clockwise. The reverse was the case for negative angular shifts.

The experiment started with a short trial in which participants were asked to turn the crank in order to check whether the writing pad worked properly and to allow participants to familiarize themselves with the apparatus. After that participants read the instructions which stated that the task was to coordinate the visual feedback of circular hand movements with a circling stimulus in four different patterns on the screen. They were explained that those patterns differed with respect to whether the visual feedback trajectory should be rotating in the same or in the opposite direction of the stimulus trajectory, and whether the stimulus dot and visual feedback dot should be on same or on opposite positions of the respective circles. Opposite meaning for example that when the dot of the stimulus trajectory was in the highest position of the stimulus circle, the dot of the visual feedback trajectory should be in the lowest position of the visual feedback circle. To illustrate those patterns, they then saw demonstrations of the four patterns they were asked to produce in visual space. The demonstration consisted of two dots in the positions of the stimulus dot and visual feedback dot, moving in the respective patterns. Participants had the opportunity to ask questions in the instruction phase as well as later prior to each trial, as the experimenter was present during the whole experiment.

After that, the procedure was the same for every trial. At first a two-word instruction for the next trial appeared on the screen, defining the stimulus-visual feedback relation (in terms of rotation direction and y-direction). Participants started trials themselves by pressing the space bar on a keyboard with their left hand. As soon as the space bar was pressed the stimulus dot appeared at the rightmost position of the stimulus trajectory and started moving. The stimulus dot increased its speed every 10 circles by 0.2 Hz (from 0.8 to 1.2 Hz, one trial thus consisted of all three speeds). Each trial lasted 30.83 s. The four visual patterns were blocked. The order of visual pattern blocks was randomized for each participant. Within each visual pattern block each of the eight angular shifts was presented in one block for six trials, the order of angular shift blocks was randomized. Thus, altogether 192 trials were performed (4 patterns × 8 angular shifts × 6 trials). It took participants between 2 h and 2 h 30 min to complete an experimental session. The duration of the experimental sessions varied, as participants had the opportunity to take breaks between trials.

### DATA ANALYSIS

**FIGURE 2:**
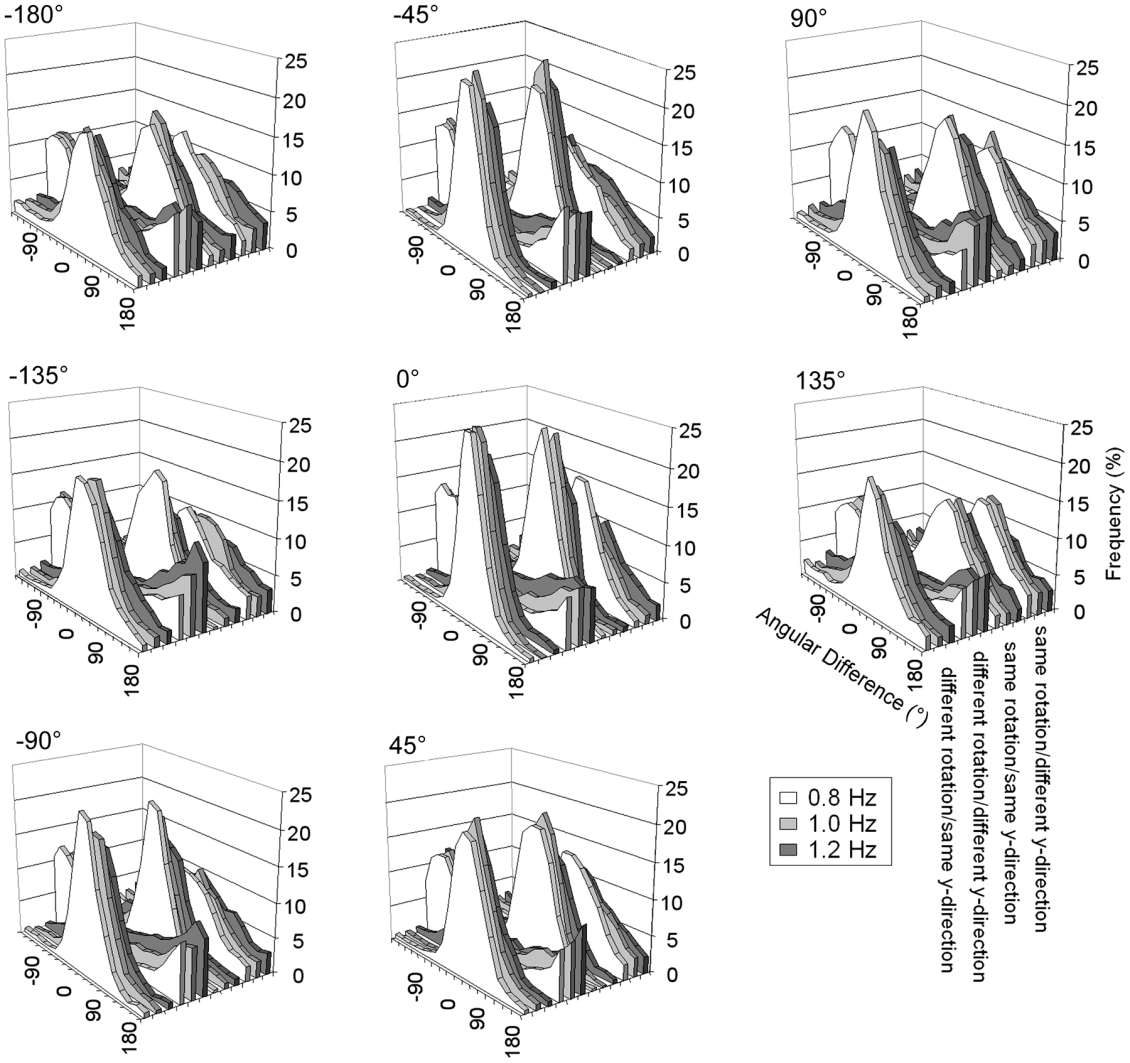
**Frequency distributions (in %) of the angular differences between the ideal angle and the observed angle depending on instructed pattern and visual speed, separately for each angular shift**.

Because we were interested in performance after participants had adjusted to a certain transformation, we excluded the first trial of each condition from analysis, as this was regarded a training trial. Further, we excluded the first three circles of every speed level, to allow time for adaptation to the new speed requirements. For the remaining data we calculated the angular difference by subtracting the ideal position of the visual feedback from the actual position of the visual feedback (see **Figure 2**). Because the shortest distance between the two points was used, the angular difference cannot be smaller -180° or larger than 180°.

Based on the angular differences, we calculated the percentage of time participants spent in the instructed mode (IM; angular differences between -45 and 45°) and the opposite mode (OM; angular difference smaller than -135° or larger than 135°; see Dietrich et al., 2012; Rieger et al., 2014 for a similar procedure calculating IM and OM). The expected value for these variables is 25% (random performance). We also calculated the spatial constant error (CE), a signed value indicating the average angular difference between the ideal and the actual angle, which indicates whether participants are in lead of or lag behind the stimulus. We also calculated the temporal CE. The data patterns of the spatial and temporal CE were very similar (as they are related in our task). We therefore decided to report the spatial CE only.

Because participants were not able to perform the instructed pattern in the different rotation/different y-direction condition (see **Figure 2** and analysis below), but rather fell into a different rotation/same y-direction pattern, we did not include this condition in the analysis in which we investigated the effects of the magnitude of the angular shift transformation on performance. Rather, we calculated ANOVAs with the factors Coordination Pattern (different rotation/same y-direction, same rotation/same y-direction, and same rotation/different y-direction), Angular Shift (-180, -135, -90, -45, 0, 45, 90, and 135°), and Speed (0.8, 1.0, and 1.2 Hz). Because the factor speed did not result in switches to other patterns (performance only declined with faster speeds), we do not report any effects in which this factor is involved.

For the investigation of the roles of movement-to-stimulus-coordination and vision-to-stimulus-coordination only the angular shift 180° in comparison to the angular shift 0° is of interest. With 180° angular shift, performance in the same y-direction condition may suffer, not only because visual feedback and stimulus have different y-directions, but also because movement and stimulus have different y-directions. However, performance in the different y-direction condition may profit, because movement and stimulus have the same y-direction. Thus, one may expect (a) that differences between the same and the different y-direction conditions are smaller with 180° angular shift than with 0° angular shift and (b) better performance in the different y-direction condition with 180° angular shift than 0° angular shift. To investigate this we performed an ANOVA with the factors Rotation Direction (same, different), Y-direction (same, different), Shift (0 and 180°), and Speed (0.8, 1.0, and 1.2 Hz) on IM. In this analysis we were only interested in interactions involving the factors y-direction and angular shift.

If Mauchly’s test indicated that the assumption of sphericity was violated we report Greenhouse–Geisser corrected *F*-values and *p*-values, and Greenhouse–Geisser’ε. *Post hoc* comparisons were conducted using *t*-tests. The significance level for *post hoc* tests was corrected using the Holm–Šídák procedure. Where appropriate exact, minimum (*p*min) and/or maximum (*p*max) *p*-values are reported.

## RESULTS

### ACCURACY OF PERFORMANCE: INSTRUCTED MODE

In a first step IM and OM were compared to chance in each condition. This analysis indicated that participants tended to be most frequently in the IM in all but the different rotation/different y-direction condition. In the different rotation/different y-direction condition IM was not above chance even with no transformation (0° angular shift) but rather below chance (*p*max = 0.005). OM was above chance in this condition (all *p* < 0.001). A similar pattern of results was observed in Dietrich et al. (2012). Thus participants produced predominantly a different rotation/same y-direction pattern when they were instructed to produce a different rotation/different y-direction pattern (see **Figure 2**).

**FIGURE 3:**
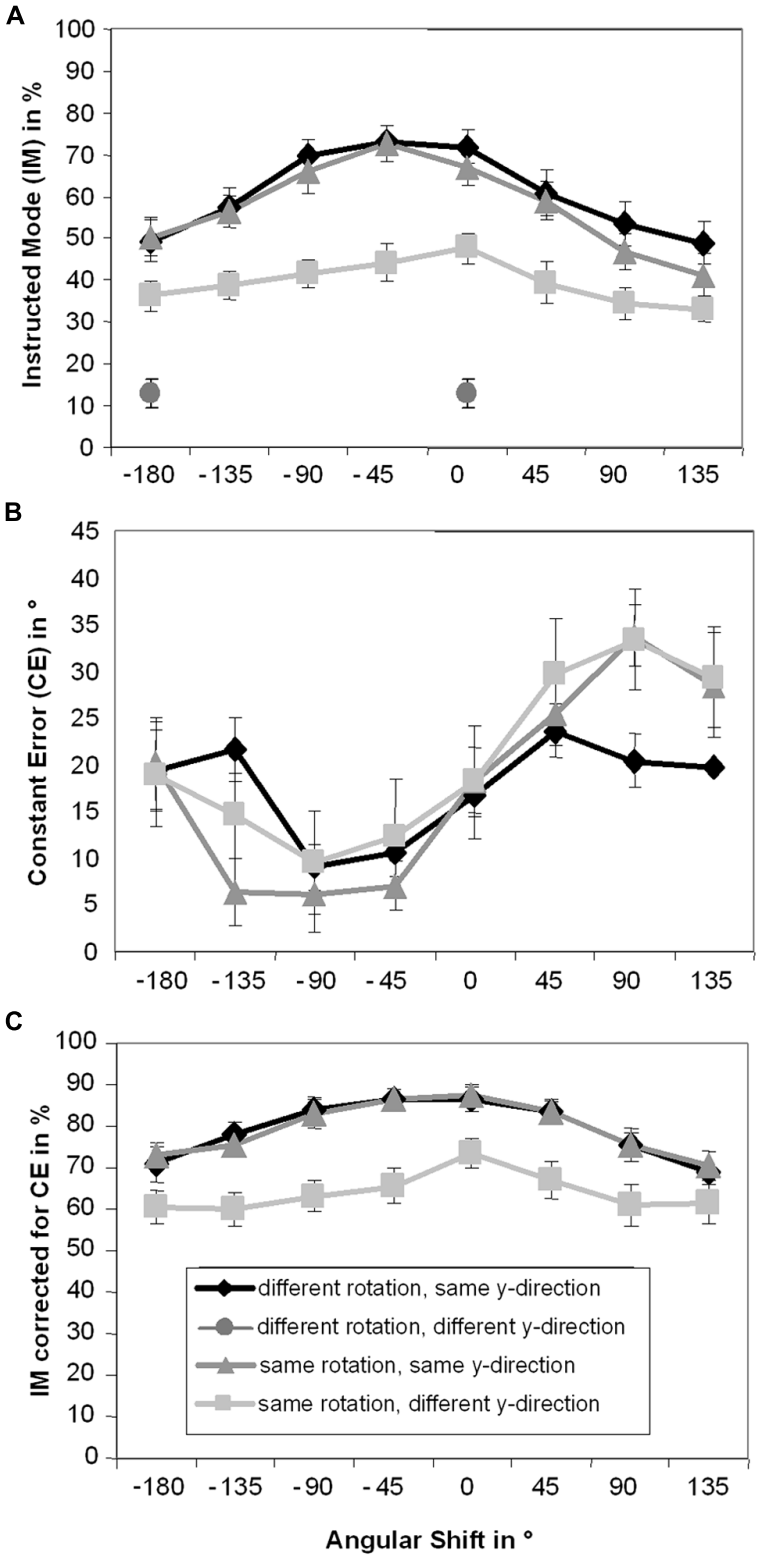
**Means and standard errors for Instructed Mode (A), Constant Error **(B)**, and Instructed Mode calculated using Constant Error **(C)** depending on instructed pattern and angular shift.** For the different rotation/different y-direction condition only the values of 0° angular shift and -180° angular shift are depicted.

Results for IM are depicted in **Figure 3A**. A significant main effect of Pattern, *F*(2,30) = 52.66, *p* < 0.001, $\eta_{p}^{2}$ = 0.78, indicated that IM was significantly lower in the same rotation/different y-direction condition (*M* = 39.4%) than in the other two patterns (different rotation/same y-direction: *M* = 60.7%, same rotation/same y-direction: *M* = 57.5%, both *p* < 0.001). IM in did not significantly differ between the latter two patterns (*p* = 0.11). A significant main effect of Angular Shift, *F*(7,105) = 42.38, *p* < 0.001, $\eta_{p}^{2}$ = 0.74, was also observed. IMs were highest with 0° (*M* = 62.2%) and -45° (*M* = 63.4%) angular shifts, which were not significantly different from each other (*p* = 0.55). IM with 0° angular shift also did not significantly differ from IM with -90° angular shift (*M* = 59.1%, *p* = 0.15), however, IM with -45° angular shift was significantly higher than IM with -90° angular shift (*p* = 0.017). IM with 0° angular shift was significantly higher than IM with all other shifts (*p*max = 0.001). In all other conditions IM successively decreased the greater the angular shift diverged from 0° (-180°: *M* = 45.4%, -135°: *M* = 50.9%, 45°: *M* = 53.1%, 90°: *M* = 45.0%, and 135°: *M* = 41.3%, *p*min < 0.001, *p*max = 0.022).

The decline in IM around 0° angular shift was asymmetric: performance was lower with angular shifts 135° than -135°, 90° than -90°, and 45° than -45° (all *p* < 0.001). Performance was however symmetric around -45° angular shift, i.e., was not significantly different between angular shifts -90 and 0°, -135 and 45°, and -180 and 90° (*p*min = 0.13), and lowest with 135° angular shift (*p*min < 0.001, *p*max = 0.022). The significant interaction between Angular Shift and Pattern, *F*(14,210) = 3.26, *p* = 0.006, $\eta_{p}^{2}$ = 0.18, ε = 0.43, slightly modified this pattern. In the same rotation/different y-direction condition successive angular shifts did not significantly differ from each other (*p*min = 0.026 in 0 vs. 45° angular shifts). Still, there was some indication of a decline in IM the larger the angular shifts were: most (but not all) shifts that were further apart than one step significantly differed from each other (*p*min < 0.001, *p*max = 0.4). The observation of less pronounced decline in IM with higher shifts in the same rotation/different y-direction condition may be due to a floor effect, as performance in this condition was worse than in the other two conditions, leading to less pronounced differences between different angular shifts.

The ANOVA to investigate whether we find subtle effects of movement to stimulus coordination showed significant interactions between Y-direction and Angular Shift, *F*(1,15) = 23.35, *p* < 0.001, $\eta_{p}^{2}$ = 0.61, and Y-direction, Angular Shift, and Rotation, *F*(1,15) = 14.62, *p* = 0.002, $\eta_{p}^{2}$ = 0.49. The difference between same and different y-directions in the different rotation condition was higher with 0° angular shift (*M* = 59.7%) than with -180° angular shift (*M* = 35.7%, *p* < 0.001). However, in the different-y-direction condition performance was not better with -180° angular shift than with 0° angular shift (*p* = 0.29). In the same rotation condition the difference between same and different y-direction conditions was not significantly different between 0° angular shift (*M* = 19.4%) and -180° angular shift (*M* = 14.1%, *p* = 0.24).

### LEAD/LAG: CONSTANT ERROR

The results for CE are depicted in **Figure 3B**. With 0° angular shift CE was 17.8°, which was significantly higher than 0° (*p* < 0.001), indicating that it may be the default mode of participants to be in advance of the stimulus. The main effect of Pattern was not significant, *F*(2,30) = 0.39, *p* = 0.59, $\eta_{p}^{2}$ = 0.03, ε = 0.63. However, a significant main effect of Angular Shift, *F*(7,105) = 13.61, *p* < 0.001, $\eta_{p}^{2}$ = 0.48, was observed. With -135° angular shift (*M *= 14.2°) and -180° angular shift (*M *= 19.5°) CE did not significantly differ from 0° angular shift (*p* = 0.13 and *p* = 0.67, respectively). With other negative angular shifts participants were significantly less in advance of the stimulus (-90°: *M *= 8.3°, -45°: *M *= 10.0, *p*max = 0.005) with positive shifts participants were significantly more in advance of the stimulus (45°: *M *= 26.3°, 90°: *M *= 29.2°, 135°: 25.8°, *p*max = 0.019) than with 0° angular shift. The interaction between Pattern and Angular Shift, *F*(14,210) = 2.06, *p* = 0.08, $\eta_{p}^{2}$ = 0.12, ε = 0.35, did not reach significance.

### CONTROL ANALYSES: IM CALCULATED USING MEAN CE

One may argue that variations in IM are due to systematic variations in CE. Because IM was calculated by using CE values within ±45° around the ideal position, it may be that when the mean CE is not 0, parts of the distribution around it are systematically not used in the calculation of IM. To rule out this possibility, we recalculated IM, using a window around participants’ mean CE ±45° for each condition. The results for IM calculated using mean CE are depicted in the **Figure 3C**. Overall, IM calculated using mean CE (*M* = 74.2%) was significantly higher than IM calculated using the ideal position (*M* = 52.5%, *p* < 0.001).

The ANOVA on IM calculated using mean CE only revealed significant main effects of Pattern, *F*(2,30) = 40.7, *p* < 0.001, $\eta_{p}^{2}$ = 0.73, ε = 0.68, and Angular Shift, *F*(7,105) = 25.9, *p* < 0.001, $\eta_{p}^{2}$ = 0.63, ε = 0.57, but no significant interaction between Pattern and Angular Shift, *F*(14,210) = 1.5, *p* = 0.21, $\eta_{p}^{2}$ = 0.09, ε = 0.41. Again, IM did not significantly differ between the different rotation/same y-direction condition (*M* = 79.2%) and the same rotation/same y-direction condition (*M* = 79.3%, *p* = 0.95). In those two conditions IM was higher than in the same rotation/different y-direction condition (*M* = 64.0%, both *p* < 0.001).

Instructed mode was significantly higher with 0° angular shift (*M* = 82.5%) than with all other angular shifts apart from -45° angular shift (*M *= 79.7%, *p* = 0.08, others *p*max = 0.003). IM again successively decreased the greater the angular shift diverged from 0° (-180°: *M* = 68.0%; -135°: *M* = 71.1%, -90°: *M* = 76.8%, 45°: *M* = 77.8%, 90°: *M* = 70.7%, and 135°: *M* = 66.9%, *p*max = 0.025, apart from 45 vs. 90°, *p* = 0.06 and 135 vs. -180°, *p* = 0.49). The decline in IM around the 0° angular shift was again asymmetric: performance was lower with angular shifts 135° than -135° (*p* = 0.001), and 90° than -90° (*p* = 0.013), but not 45° than -45° (*p* = 0.36). Performance was however symmetric around the middle of the angular shifts of -45 and 0°, i.e., was not significantly different between angular shifts -90 and 45° (*p* = 0.53), -135 and 90° (*p* = 0.78), and -180 and 135° (*p* = 0.49).

## DISCUSSION

To investigate how the perceptual-motor system deals with shift transformations in unimanual circling we asked participants to coordinate the visual feedback of their hand movement with a continuously circling stimulus in order to produce four different patterns in visual space. The patterns they were asked to produce consisted of same and different rotations of stimulus and visual feedback, either in same or different y-directions. The movement-visual feedback relation was manipulated using eight angular shifts: (-180, -135, -90, -45, 0, 45, 90, and 135°). Participants were not able to perform the different rotation/different y-direction pattern. Instead they fell into the different rotation/same y-direction pattern (defined in terms of visual space). The different rotation/same y-direction pattern and the same rotation/same y-direction pattern were performed equally well, performance was worse in the same rotation/different y-direction pattern. Best performance was observed with 0° angular shift and with -45° angular shift. Performance declined with increasing shift, the 180° angular shift condition was no exception. The decline was symmetric around -45°/between -45 and 0° angular shift. Participants did not fully take the angular shifts into account: when angular shifts were negative, the visual feedback was less in advance of the stimulus than with 0° angular shift, and when angular shifts were positive, the visual feedback more in advance of the stimulus than with 0° angular shift. However, this diminished with higher angular shifts, the CEs of -135° angular shift and -180° angular shift did not significantly differ from the CE of 0° angular shift. No clear indication of movement-to-stimulus coordination in the different y-direction conditions with -180° angular shifts was observed.

Similar to Dietrich et al. (2012) the relative difficulty of the coordinative patterns resembles results from bimanual coordination studies (e.g., Swinnen et al., 1997). Participants were not able to produce the different rotation/different y-direction pattern in visual space, but tended to produce the different rotation/same y-direction pattern. This is also the most difficult of the four patterns in bimanual coordination (Swinnen et al., 1997), and participants tend to fall into the easier coordination pattern (Semjen et al., 1995). Further, same y-directions of stimulus and visual feedback were advantageous for performance in comparison to different y-directions between stimulus and visual feedback, an observation which has also been made concerning the y-directions of the two hands in bimanual coordination (e.g., Swinnen et al., 1997). This suggests that the principles by which bimanual and unimanual coordination are governed are similar (see also Buekers et al., 2000; Dietrich et al., 2012). Similar results are also obtained when two people perform coordination patterns together (Schmidt et al., 1990). Therefore, the stimulus circle in the present task may have been represented in a way similar to the way another person performing a movement is represented.

However, in contrast to studies on bimanual coordination, we observed no difference in performance between the different rotation/same y-direction condition (which results in a mirror symmetric pattern on the screen) and the same rotation/same y-direction condition (which results in a parallel pattern on the screen). A similar observation has been made by Dietrich et al. (2012). In bimanual coordination mirror symmetric movements are generally associated with better and more stable performance than parallel movements (e.g., Swinnen et al., 1997; Mechsner et al., 2001; Tseng et al., 2006). Swinnen et al. (1997; see also Kelso, 1984) argue that the performance advantage of mirror symmetric movements is due coactivation of the same (homologous) muscles of the two limbs. Alternatively, or in addition, it has been argued that the specification of equal movement parameters for both limbs plays a role for this effect (Heuer, 1993; Cardoso de Oliveira, 2002). Because we used a unimanual task, coactivation of homologous muscles cannot occur, and movement parameters are specified only for one hand. The similar performance in the different rotation and the same rotation conditions (with same y-directions between stimulus and visual feedback) can thus be attributed to the absence of such motor constraints. In terms of perceptual constraints, different rotations and same rotations may be equally difficult. Indeed, participants sometimes even prefer parallel motions over symmetric motions when they have to rely on visual feedback (Alaerts et al., 2007).

Performance patterns in visual space were similar under all angular shift conditions, indicating that vision-to-event coordination dominated performance. This is in accordance with unimanual and bimanual coordination research showing dominance of visual information over proprioceptive or kinesthetic information (e.g., Mechsner et al., 2001; Bogaerts et al., 2003; Roerdink et al., 2005) and also research on tool transformations using other tasks (Sutter, 2007; Sutter et al., 2011). The dominance of vision might be due to the quality of visual feedback: visual information is less noisy than proprioceptive information, and visual feedback is usually readily available (Wilson et al., 2005a,b). Another reason for the dominance of vision rather than proprioception may be that vision is more distal than proprioception. It has been suggested that distal rather than proximal movement consequences provide the main reference frame for movement planning and execution (Prinz, 1997; Hommel et al., 2001). Therefore movement representation in the external world may be on the highest level of a hierarchical structure of movement planning and execution (Rieger et al., 2005). However, given that the task was defined in terms of the stimulus-visual feedback relation, the dominance of vision-to-event coordination over movement-to-event coordination may not be surprising.

Nevertheless, performance with 0° angular shift (regular visual feedback) and -45° angular shift was more accurate than performance with other shifts. Thus, producing visual patterns is not sufficient for coordination, as the patterns were the same in all shift conditions. If only the visual pattern had mattered for performance, the transformations of the movement-visual feedback relation should have had no effect on performance. Thus, in accordance with other studies (Roerdink et al., 2005; Kunde et al., 2007; Massen and Prinz, 2007; Lepper et al., 2008; Sülzenbrück and Heuer, 2010; Dietrich et al., 2012; Rieger et al., 2014) there are costs when a transformation is present. Importantly, performance declined with increasing shift. Thus, the magnitude of the transformation mattered. Larger shifts may have been experienced as more incongruent and therefore more difficult. This is in accordance with findings showing that the likelihood of consciously detecting a transformation depends on its magnitude (Fourneret et al., 2002; Knoblich and Kircher, 2004; Rieger et al., 2014). Performance with small negative angular shifts (i.e., -45°) was comparable to performance with no angular shift, and the performance decline was symmetrical around an angular shift of less than 0°. Even though the asymmetry around 0° diminished slightly when performance accuracy was corrected for the CE, it was still present. Thus, it was more advantageous when the hand was in advance of the visual feedback than when it was behind the visual feedback.

The 180° angular shift condition was no exception to the decline in performance with larger angular shifts. We had thought that the difficulty of the movement-visual feedback relation might follow similar principles as the difficulty of hand–hand relations in bimanual coordination. In bimanual coordination moving in opposite y-directions is easier than moving at other phase relationships between the hands (apart from moving in the same y-direction; Tomatsu and Ohtsuki, 2005). However, such an effect was not observed. Even in the different y-direction condition the 180° angular shift condition was not beneficial. Here, movements and stimulus move in the same y-directions which may have been used to benefit performance. The observation that no movement-to-stimulus coordination occurred in the same rotation condition is in accordance with a previous study in which a similar task was used (Dietrich et al., 2012). However, previously it was observed that movement-to-stimulus coordination occurred in the different rotation condition (180° angular shift resulted in better performance than 0° angular shift), which indicated that proprioceptive information from the hand was used to aid performance. This was not the case in the present study. Even though the difference between same and different y-directions was larger with 0° angular shift than 180° angular shift, performance in the 180° angular shift condition was not better than in the 0° angular shift condition, which speaks against movement-to-stimulus coordination. It was particularly surprising that this effect was not found, because vision-to-stimulus coordination was difficult in the different rotation/different y-direction condition. Performance was below chance. Movement-to-stimulus coordination may have been used to improve performance. How can the differences between the two studies (previously we found evidence for movement-to-event coordination, here we do not) be explained? In the present study we used several shifts, but only one shift was used in the previous study. The use of several shifts may have made it harder for participants to detect that they can make use of movement-to-stimulus coordination, as it could not be used in most shifts of the experiment. Thus, the experimental context may have prevented participants from applying this strategy. The failure to detect that such a strategy is possible most likely occurred, because proprioceptive information may not have been perceived with a high spatial accuracy. In similar tasks participants are not very good in knowing their actual hand positions (Fourneret and Jeannerod, 1998; Rieger et al., 2014).

Overall visual feedback was more likely to be in lead of the stimulus, which was also the case when no transformation was present (0° angular shift). This may be due to participants’ use of the dominant hand in the task. The dominant hand shows a slight lead over the non-dominant hand when coordinating symmetrical movements in bimanual coordination (Treffner and Turvey, 1995). This seems to be due to attentional factors, because the lead of the dominant hand disappears when attention is directed to the non-dominant hand (Amazeen et al., 1997). Participants probably paid more attention to the visual feedback than the stimulus.

The CE was systematically influenced by the magnitude of the transformation. Participants did not fully take the transformation into account: when the angular shifts were negative the visual feedback was less in advance of the stimulus than in 0° angular shift, and when angular shifts were positive the visual feedback was more in advance of the stimulus than in 0° angular shift. These results are also in accordance with results on shift transformations in bimanual coordination (Tomatsu and Ohtsuki, 2005). Tomatsu and Ohtsuki (2005) asked participants to perform circling movements with the two hands (one clockwise and one counterclockwise) in four different relative phases between the hands: 0, 90, 180 and 270°. In a transformed feedback condition visual feedback of the right hand was shifted such that performing those patterns in movement space resulted in mirror symmetry in visual space. Thus, as in our task, the shifts were present in movement space but not in visual space. Similar to our results, the right hand was in advance of the ideal angle in the 90° shift condition and lagged behind the ideal angle in the 270° shift condition (comparable to -90° angular shift in our study). This indicated that movements tend to shift toward 0° phase relations. Thus, like in our study, the transformation was not sufficiently taken into account. The results are also in accordance with a previous study in which gain transformations were applied in a unimanual coordination task (Rieger et al., 2014): with high gains the visual feedback was in advance of the stimulus suggesting that the magnitude of the gain might be underestimated. With low gains the visual feedback lagged behind the stimulus, suggesting that the magnitude of the gain might be overestimated. Altogether, the results suggest that the magnitude of a transformation is insufficiently taken into account.

However, the CE with -135° angular shift and -180° angular did not significantly differ from the CE of 0° angular shift, indicating that the transformation was accounted for in more extreme shifts. This is in contrast to the results on performance accuracy, which indicate that the 180° angular shift condition was not performed better than other shifts. Thus, in terms of *how* the 180° angular shift condition and a shift close to it are performed, i.e., the applied strategy, performance resembles the 0° angular shift condition. Even though the 180° angular shift is the most drastic one (visual feedback and movement are a maximal distance apart), applying the same strategy with 0° angular shift might be easier than at smaller angular shifts because the hand is exactly opposite of the visual feedback.

It is assumed that the nervous system controls movements using internal models (Wolpert and Flanagan, 2001) Inverse models choose appropriate motor commands for desired action goals and forward models predict the sensory consequences of motor commands. These predictions can refer to bodily consequences (e.g., kinesthesis and proprioception of the hand movement) and to consequences in external space (e.g., visual feedback). When a movement is transformed as in tool use external consequences do not coincide with the bodily consequences (Wolpert and Flanagan, 2001). In tool use people develop internal models of/adapt internal models to the tool transformation in order to choose motor commands and to make predictions about resulting sensory consequences which take the tool transformation into account (Imamizu et al., 2003, 2007; Rieger et al., 2008; Sülzenbrück and Heuer, 2012). Our data suggest that in the present task internal models do not fully take the shift transformation into account. If that were the case, the case, the CE should not differ between the different angular shifts. However, in accordance with previous studies (Tomatsu and Ohtsuki, 2005; Rieger et al., 2014) the transformation is represented as smaller than it actually is, resulting in imprecision. This is also in accordance with findings that the nervous system does not necessarily completely adapt to observed errors (Wei and Kording, 2009).

The present results have implications for the use of tools with shift transformations. First, such movements are more difficult to perform than untransformed movements. Thus, there are limits to the dominance of visual feedback in controlling actions involving tool transformations (see also Sutter et al., 2013). Second, the representation of the transformation in internal models can be flawed. It is important to note that the performance decrements and flaws in the representation of the transformation were observed even though initial adaptation to gains and speeds was excluded from data analysis. However, with extended practice further adaptation processes may take place. Also, telling participants about the exact nature of the shift transformation may be beneficial for performance, as it has been shown that cueing the transformation is in some cases more beneficial than cueing the action goal in tool actions (Massen and Prinz, 2007; Massen and Sattler, 2012). Knowledge of the nature of the transformation may result in participants consciously choosing strategies to aid performance.

We conclude that the mere presence of a transformation has a negative impact on performance. The representation of the transformation may be flawed. When designing machines or tools that involve transformations between movements and their external consequences, this should be taken into account.

## Conflict of Interest Statement

The authors declare that the research was conducted in the absence of any commercial or financial relationships that could be construed as a potential conflict of interest.
